# False Impressions

**Published:** 1889-03-23

**Authors:** Caroline Fothergill

**Affiliations:** Author of "An Enthusiast," "A Voice in the Wilderness," etc.


### FALSE IMPRESSIONS.

By CAROLINE FOTHERGILL, Author of "An Enthusiast," "A Voice in the Wilderness," etc.

(Continued from p. 384.)

CHAPTER XXIV.—TELLING EDITH.

GEORGE did not at once make known his engagement at home. He was reticent, both by nature and habit, and accustomed to keep both his joys and griefs to himself. He knew, too, in spite of what Edith had once said, that his sister would not be pleased. More than once he had thought of that conversation with Edith, and had wondered what she meant by saying that Mrs. Lester had been very kind and would like his marriage. Still, his sister must be told, and told soon, that was due to her; but the person whom he first informed of the impending change in his life was his friend Dr. Temple, and to him he wrote a long letter in which he made revelations so frank as to considerably startle his friend.

"I've been a solemn prig and a conceited ass," he wrote. "Till now I have been only half alive. Miss Stanford has completely changed the world for me; new hopes and beliefs are growing up in me until I scarcely know myself. I have been born again. There is this excuse for me; she is the only woman of her kind that exists, and until I met her it was only in nature that my eyes should be blinded so that I could not see. Now, as I said before, I am a new man and I am going to begin life afresh. It will not be a long engagement, and for many reasons I intend you to be best man, so you may make up your mind to lose a day's practise."

Dr. Temple's reply to this was characteristic. He wrote quietly, but with a veiled sarcasm which might have touched Mr. Murray's temper in less happy days, but which now only caused him to laugh gleefully.

When Beatrice returned to Mayfield, it became necessary to tell his sister of his engagement, and with this purpose he sought her in her sitting-room. She was alone, and he began at once with—

"Can you spare me a few minutes, Frances? I have something of importance to tell you."

Somehow—by a woman's instinct, no doubt, she guessed what was coming and that her plan had failed. Her heart seemed to stop beating. She could not speak. She only looked at him in silent expectation.

"I am going to be married," he said abruptly.

"Indeed! to whom?" she forced herself to ask, for her tongue seemed to cleave to the roof of her mouth.

"To Miss Stanford," he answered, in a tone almost as cold and restrained as her own.

"Miss Stanford!" she repeated blankly, as she saw that her worst fears were realised; and then such a tempest of anger rose in her heart and filled it to overflowing that for the moment she could say no more. She forgot Edith, she thought of her no more than if she did not exist. Her plan of telling her brother that Edith loved him, which she was determined should be her last card, to be produced as a last resource, went completely out of her head. Her one thought was for herself, her own discomforture and defeat. She did not even bestow a thought upon what this intention of George's would mean to Edith. She heard George speaking, but was too excited to know what he was saying. When a few moments had gone by she heard him say—

"I scarcely ventured to hope Miss Stanford would accept me, now she has done so I can hardly realise my immense happiness."

At this she raised her head; she had come to herself again and was smarting and burning with anger. Words stung her tongue for utterance and she could not restrain them.

"Ah!" she said, in a tone of contempt which came strangely from her to her brother, the one person whom she had always treated with deference, "you are as blind as other men when you are in love. Everyone else has seen from the beginning that she was doing her very best to catch you."

He turned towards her white with wrath, his eyes blazing, and with a look which, at any other time, would have made her wish to sink into the ground; but she was past that now, and before he had recovered from his astonishment sufficiently to speak, she went boldly on with a laugh of scorn—

"Yes; both she and her dear friend, Mrs. Ledward wanted you, and, at one time, it seemed about an even chance between them. I suppose they have arranged that, on the whole, it would be best for you to marry Miss Stanford. No doubt Mrs. Ledward will have her own quarters here, though I must turn out. It is a queer household, and if what one hears about the way in which Miss Stanford has spent the last two months be true, she has very queer tastes and occupations. In my youth ladies did not fill their houses with people picked up out of the streets, and call them friends, and eat at the same table with them. A queer woman for a Murray to marry, but she has money and a bold face."

Her words had come in a torrent, impossible for George to stem their flow, but now she paused for breath, and he went up to her and laid his hand upon her arm.

"Frances," he said, in a curious voice, which sounded not like his own—"you are a woman, and my sister, but that does not entitle you to give free license to your tongue. You must be mad, simply mad, to speak as you have done. If you are not mad, you are the most evil-minded woman that ever lived. But I prefer to think that you do not know what you are saying."

"Of course I am mad," was her reply; "both mad and wicked, if you prefer it. From the beginning of the world it has been the fate of tellers of the truth to be considered either mad or bad, or both. Every word I said was true, and you would have been the first to see it in the case of anyone except yourself. I repeat, she has ——"

"Silence!" he said, arresting her speech half-way. "One word more and I shall forget myself. You are angry and I expected it, but I did not expect a scene like this, and I will not endure it. The civilities it will be necessary to pay to Miss Stanford you shall pay her, but beyond that our intercourse ceases; we can no longer be brother and sister. I will only tell you that our engagement will not be a long one, and you can make the necessary arrangements for removing your things as early as you please."

He went out of the room as he spoke, leaving Mrs. Lester almost beside herself with anger and dismay. Never had plan failed as miserably as hers had done. It had been trampled underfoot—nay, worse, ignored. It had never entered George's head that any plan would ever be laid against him, and he had never behaved as though he were in fear of one. She had played her cards badly indeed, for she had completely estranged her brother. She knew him too well to suppose he would ever forgive the words she had said about Beatrice. Suddenly she thought of Edith, and her anger turned against her. Why had she not managed her affair better? She had had ample time and opportunity, if she had only made a proper use of both. In ten minutes she had persuaded herself that the whole catastrophe was due to what she called "Edith's childishness and imbecility." She resolved that when she saw her niece she would speak to her as she deserved.

George went downstairs feeling outraged. He had never seen his sister like this before, and he wondered how it was that the natural coarseness and evilness of her disposition should have slumbered so long before bursting out. He had always known she had a violent temper, but he had never supposed she had it in her to bring about such a scene as that through which he had just passed. What in the world had Edith meant by saying her aunt would like his marriage? He intended to go to his study, but passing the drawing-room door, which stood open, he saw Edith sitting within, and paused.

It may seem strange, but after this violent and disgusting scene with his sister, he felt a strong desire for a woman's society and sympathy. There was Edith, the very woman he wanted; gentle, and, to a certain extent, comprehending. He could ask her, too, what she had meant by the mysterious utterance which he had just remembered. On the spur of the moment he took a step forward into the room.

Edith looked up in some surprise. It was seldom he came into this room in the morning, and she at once felt glad that she happened at that moment to be reading a book which he had himself given her, expressing at the same time a desire that she should make herself acquainted with it. She expected that he would make some remark upon her occupation, but he did not, he only sat down near her and looked at her. The interview with his sister had left its mark upon his face, and Edith saw at once that something had happened. For some little time she dared not ask what it was, but sat in suspense waiting for him to speak. His silence lasted so long, however, that when she could bear it no longer, she plucked up courage to ask—

"Is anything the matter, Mr. Murray ?"

"Yes," he answered, rousing himself. "I have just been having a conversation, a very disagreeable conversation with your aunt. Edith, do you remember my telling you that I was thinking of getting married, and that she would not like it, and you said she would. What made you imagine it could possibly be ?"

"She told me so," said Edith, blushing.

"No ; you are mistaken, you misunderstood her, she must have been speaking of something else. I have just been telling her of my intention, and she is very angry, and her anger is of a very dreadful kind ; full of hatred and malice, taking the form of vulgar and violent abuse of the person with whom she is angry. We have just had a very painful scene, and you will have to know it sometime ; we have ceased to be brother and sister. She will leave this house as soon as she can arrange to do so."

He spoke coldly, for it cost him a great effort to say as much as this even to Edith. To expose his family quarrels to any except those concerned was hateful to him, but Edith would have to know, and he preferred telling her himself.

Edith heard him in amazement. She did not know what to think. Why had her aunt changed her mind ?

"I am very sorry," she said at last, puzzled and shocked and almost bewildered.

"Yes," went on George, "it has been a great shock, and her anger is thoroughly unreasonable. Why should I not marry if I wish ? Why is it that women expect just the one man with whom they are concerned never to marry, although they rejoice over the marriages of other men ? However, that is not to the point. I am going to marry and—Edith, I believe you are really fond of me."

"Oh, yes, of course ;" she answered with a little gasp, not looking at him, and rather shrinking away, with varying colour, in a way which would have puzzled George at any other time.

"And you will not be angry at my marriage ?"

He smiled as he spoke, to him the idea of her being angry was ludicrous. But to her the smile seemed one of affection and encouragement, and she smiled too as she answered more boldly, although still tremulously.

"It will make me very happy. How could you think it would not ?"

"I believe it will ! I believe it will !" he said. "I intend it to. At present your aunt tyrannises over you a little, but before long I intend to alter all that. When we are married——"

In his love-lorn state, persons could be quite clearly indicated without names, and he would have been amazed if any one had told him he had never mentioned Beatrice to Edith in connexion with his matrimonial plans. He was therefore considerably astonished when, as he spoke the last words, Edith suddenly pushed her chair further from him, and hid her face in her hands, while, trembling from head to foot, she stammered out :

"Oh, Mr. Murray, you should not take it so for granted. I never——"

"My dear Edith !" he said, looking at her, and lost in amazement. "What is the matter ? Why do you look like this ? Are you ill ? What have I said to cause you such distress ? Does your womanhood feel hurt because you think I have taken Miss Stanford's consent for granted ? You are mis——"

He stopped, for Edith had risen, and was standing before him pale and breathless, her eyes fixed upon his face. She seemed trying vainly to say something, and he rose too, and, alarmed at her appearance, said :

"Edith, what is the matter ?"

"Miss Stanford !" she said at last. "Why do you speak of Miss Stanford ?"

"My dear child," he said, half-impatiently, for he felt as if a second scene would be too much for him, "where are your wits ? Miss Stanford is the lady I am going to marry. You said you knew."

She made no answer. Her lips moved, but no words came. She only stood looking at him with an expression which at first puzzled him ; and then, suddenly—how, he never knew—the truth came like a flash into his mind, and he took a step nearer to her, saying :

"Edith, is it possible you——"

But she swerved away from him, crying out :

"Oh ! no, no ! You must not touch me. Oh, Aunt Frances, how could you? "

She took a few trembling steps towards the door, and then her strength failed, and George just caught her before she fell to the ground.

(*To be concluded.*)

